# Biogeography of Australian Camphorosmeae and Diversification in Climatic Space and Across Arid Habitat Types

**DOI:** 10.1002/ece3.70558

**Published:** 2024-11-14

**Authors:** Jessica A. Berasategui, Anže Žerdoner Čalasan, Gudrun Kadereit

**Affiliations:** ^1^ Prinzessin Therese von Bayern Lehrstuhl für Systematik, Biodiversität & Evolution der Pflanzen Ludwig‐Maximilians‐Universität München Munich Germany; ^2^ Institute for Molecular Physiology Johannes Gutenberg‐University Mainz Mainz Germany; ^3^ Botanischer Garten München‐Nymphenburg und Botanische Staatssammlung München Staatliche Naturwissenschaftliche Sammlungen Bayerns Munich Germany

**Keywords:** ancestral reconstruction, Atlas of Living Australia, Australia, biogeography, Camphorosmeae

## Abstract

This study investigates the biogeography of the Australian Camphorosmeae (Amaranthaceae *s.l.*) lineage and how it relates to shifts in climatic niche and habitat types. Building on previous research and data resources, we integrate molecular phylogenetics, bioclimatic data and biogeographical models to deepen our understanding of the diversification and adaptation of this group across Australia's diverse landscapes in relation to palaeoclimatic changes. For 159 species representing 12 genera, georeferenced distribution points were used to define the most informative bioclimatic variables using principal component analysis. Evolutionary shifts in climatic niches and habitat types were analysed, revealing clade‐specific shifts and adaptations to different habitats and climatic conditions. Biogeographical analyses allowed us to infer ancestral areas of Camphorosmeae in Australia and relate their expansion over evolutionary time to habitat shifts. Preadaptation of this group to warm and dry habitats coupled with key periods of aridification in Australia, particularly during the Late Miocene to Pliocene, were critical in driving its diversification through migration and local adaptation to varied habitats of arid Australia. Our analyses suggest that the ‘Riverine Desert’ habitat offered suitable conditions for ancestral Australian Camphorosmeae and facilitated their early widespread dispersal in the Western and Eastern Desert. We hypothesise that early diverging lineages such as *Roycea* adapted to the later emerging ‘Desert Lake’ habitat when it spread in Western Australia during the Early Pliocene. Further, habitat type shifts occurred from ‘Riverine Desert’ to ‘Shield Plain’, ‘Karst Plain’ and to ‘Sand Desert’ also during the Pliocene and Pleistocene once these habitat types emerged. This study illustrates the complex interplay between ecological flexibility and niche conservatism in shaping the biodiversity of Australian Camphorosmeae.

## Graphical abstract

Integrating molecular phylogenetics, bioclimatic data and biogeographical models, this study explores the biogeography of Australian Camphorosmeae (Amaranthaceae s.l.), revealing clade‐specific shifts and adaptations to different habitats and climatic conditions over evolutionary time. The findings of this study highlight how key periods of aridification and existing adaptations to warm, dry habitats facilitated the diversification and widespread occurrence of Camphorosmeae in Australia, with significant habitat type shifts during the Miocene to Pleistocene epochs.

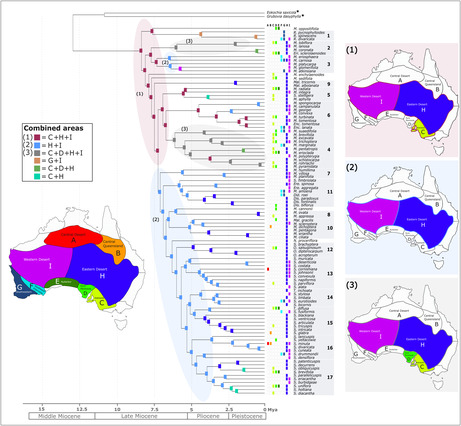

## 1 Introduction

Australia is characterised by diverse landforms, a wide variety of biogeographical regions and unique fauna and flora. While the northern regions of the continent experience tropical savannah climate with high humidity and a pronounced wet summer and dry winter season, the humidity, temperature and seasonality gradient decline along the eastern coast, which experiences a more moderate oceanic climate (Hadwen et al. 2011). On the contrary, southern and southwestern regions are under strong Mediterranean influence with high summer temperatures and a rainfall period during the winter months. The largest central portion of the continent, however, experiences a hot semi‐arid and arid climate, with annual precipitation below 500 mm and 250 mm, respectively (Hadwen et al. 2011; Pearce et al. 2010). One of the most noteworthy palaeoclimatic events in Australia's history with substantial effects on flora and fauna occurred around 33 million years ago, during the Eocene to Oligocene transition. During this period, the continent experienced significant climate cooling and increased seasonality, followed by a later phase of aridification (Byrne et al. 2008; Crisp and Cook 2013; Feakins, Warny, and DeConto 2014; Nge et al. 2020). These climatic shifts led to the spread of arid environments across much of Australia, influencing the dispersal and diversification of sclerophyllous plant species, especially those which were already preadapted to nutrient‐poor and drought‐prone conditions (Martin 2006; Crisp and Cook 2013). Around 30 million years ago, Australia had separated from Antarctica and continued to drift northwards with increased aridification leading to the transformation of previously abundant rainforest vegetation into sclerophyll woodlands. In these woodlands, the flora became dominated (in terms of biomass and impact on the environment) by a variety of plant families, including Proteaceae (*Banksia* L.f., *Grevillea* R. Br. ex Knight, and *Hakea* Schrad. & J. C. Wendl.), Myrtaceae (*Eucalyptus* L'Hér.), Fabaceae (*Acacia* Mill) and Asteraceae (*Olearia* Moench) (Dettmann and Jarzen 1998; Ladiges, Udovicic, and Nelson 2003; Crisp and Cook 2013; Jansen, Michaels, and Palmer 1991).

Australia's diverse habitats and ecosystems, spanning from the Gondwanan remnant rainforests to the much younger outback deserts, are categorised into distinct biogeographical regions. This classification guides conservation efforts, ecological research, land management and environmental policies. By identifying areas of high biodiversity, researchers can prioritise conservation efforts, study species distribution patterns and develop environmental policies. In essence, the organisation of Australia's ecosystems into biogeographical regions is a practical tool for understanding, conserving and managing the country's remarkable biodiversity and natural resources. However, this led to multiple bioregionalisations in Australia, with areas being synonymised or overlapped (Ebach 2012).

One of the most common classifications is the *Interim Biogeographic Regionalisation for Australia* (Thackway and Cresswell 1995; IBRA7), which divides Australia into 89 bioregions and 419 subregions. This classification system is based on shared characteristics such as climate, geology, landforms, vegetation and species distributions (Thackway and Cresswell 1995). Another well‐known bioregionalisation is the *Integrated Marine and Coastal Regionalisation of Australia* (IMCRA, Commonwealth of Australia 2006), which classifies marine and coastal environments into several bioregions based on oceanographic data, biological distributions and habitat types for marine conservation. The definition of bioregions or subregions, in general, is fundamental to understanding the distribution patterns of biodiversity. By defining regions based on shared ecological characteristics and species distribution patterns, these classifications enable targeted interventions to conserve biodiversity hotspots. (Ebach 2012; Ebach et al. 2015) suggested a taxonomic convention for defining and naming the phytogeographical areas of Australia. They identified several broad phytogeographical regions and subregions across the continent based on the distribution patterns of specific taxonomic groups rather than also considering geology and landforms as used in the IBRA system. Each of these regions or subregions is characterised by different ecological traits, biodiversity and geological characteristics and contributes to the overall diversity of Australia's landscapes and ecosystems. Overall, the subregions are divided into 20 phytogeographical regions, including the western, central and eastern deserts, the Great Sandy Desert Interzone, Central and Eastern Queensland, the Nullarbor, Eyre Peninsula and Adelaide areas.

Around 18% of Australia's central landscape is classified as desert, with each desert landscape having its characteristics and attributes (Geoscience Australia 1994). Additionally, there are other areas that experience desert‐like conditions due to low rainfall and high temperatures. Mabbutt (1988) described and mapped eight habitat types across arid Australia based on landforms and corresponding vegetation types (e.g., Desert Uplands, Shield Plains, Stony Desert, Karst Plain, Sand Desert, Riverine Desert, Desert Clay Plains and Desert Lakes). McDonald (2020) introduced two additional habitat types following his study of Australian chenopods (i.e., formerly classified under Chenopodiaceae, now part of the Amaranthaceae) across different arid landscapes and adjacent temperate, subtropical and coastal zones (e.g., Mesic Plain, Range, and Coast). Due to the diversity of Australia's ecosystems coupled with high isolation of the whole continent, high number of species as well as high levels of endemism are observed in contemporary Australian angiosperms. The most species‐rich Australian plant families with notable endemic lineages include Asteraceae, Cyperaceae, Ericaceae (Epacridoideae), Euphorbiaceae, Fabaceae (Mimosoideae), Goodeniaceae, Myrtaceae, and Orchidaceae, Poaceae, Proteaceae and Rutaceae, with Amaranthaceae Juss. sensu lato present in high numbers in Australian arid regions (Beadle 1981; Morley and Toelken 1983; Hnatiuk 1990; Crisp and Cook 2013; Ladiges, Udovicic, and Nelson 2003; Funk 2009; Walker et al. 2018; Morales‐Briones et al. 2021).

One of the most species‐rich Australian tribes within Amaranthaceae sensu lato is Camphorosmeae Moq. It comprises around 160 Australian species placed in 12 genera (according to Plants of the World Online; POWO 2024), with the two biggest genera being *Sclerolaena* R.Br. (79 spp.) and *Maireana* Moq. (58 spp.). Australian Camphorosmeae are widely distributed across the continent, and are found in different habitats, from coastal dunes to inland deserts. While Australia harbours the highest species diversity of this tribe, there are approximately 35 species across eight genera that occur outside of Australia. These non‐Australian species are distributed in various arid and semi‐arid regions, including the Canary Islands, parts of Eurasia (mainly in the Old World Desert Belt stretching from the Middle East to Central Asia), North America (e.g., *Neokochia* (Ulbr.) G. L. Chu and S. C. Sand.) and South Africa. These species typically inhabit arid, semi‐arid and coastal regions, often characterised by saline or sandy soils and low precipitation (Kadereit, Gotzek, and Freitag 2005; Kadereit and Freitag 2011; Hühn et al. 2024). Biogeographical and molecular evidence suggests that the non‐Australian species represent early divergent lineages of the tribe Camphorosmeae, particularly within Eurasia. The genus *Grubovia* Freitag and G. Kadereit is sister to the Australian Camphorosmeae and native to Central Asia (Kadereit and Freitag 2011; Kadereit et al. 2014). Camphorosmeae likely originated in the Old World during the Late Eocene to Early Oligocene (Kadereit and Freitag 2011). The earliest divergent lineages, such as the *Chenolea* Thunb. clade, which includes species from Eurasia, western North America and southern Africa, are thought to be remnants of older evolutionary branches that have undergone significant extinctions (Kadereit and Freitag 2011).

The Australian Camphorosmeae are remarkably well adapted to extreme environmental conditions, including saline soils and arid areas (Kadereit, Gotzek, and Freitag 2005), while presumably performing exclusively C_3_ photosynthesis (Carolin 1975; Jacobs 2001; Freitag and Kadereit 2014). These adaptations include morphological traits such as reduced leaf size, succulence and hairy or scaly surfaces, which minimise water loss (Kadereit and Freitag 2011). This lack of specialised C_4_ photosynthesis is also common in many other species‐rich Australian desert plants, such as *Acacia* (Fabaceae), *Eucalyptus* (Myrtaceae), *Eremophila* R.Br. (Scrophulariaceae) and *Ptilotus* R.Br. (Amaranthaceae), which indicates that C_4_ photosynthesis alone cannot explain the evolutionary success of some C_4_ lineages in water‐scarce conditions (Bowman and Cook 2002; Sage et al. 2007; Kattge et al. 2011). Understanding the evolutionary history and biogeographical patterns of Camphorosmeae helps to elucidate the potential mechanisms underlying the diversification and adaptation of lineages in arid ecological niches.

Molecular phylogenetic research has become an important tool to improve the understanding of evolutionary relationships and genetic diversity within this tribe, albeit initially with limited sampling and low support. The taxonomic position of the Camphorosmeae has thus changed several times (Freitag and Kadereit 2014). Cabrera, Jacobs, and Kadereit (2009) conducted the first molecular phylogenetic study for this tribe, including 71 species from all recognised Camphorosmeae genera. Cabrera's study concluded that although some morphological characters such as fruiting perianth were relevant, the molecular results did not fully support the existing taxonomy, attributing this discrepancy to incomplete lineage sorting and ongoing hybridisation within the Australian Camphorosmeae group. Subsequent biogeographical analyses suggested that the Australian Camphorosmeae migration started in the southwest of Australia via a single long‐distance dispersal event from continental Asia during the Miocene, from where this lineage expanded eastwards and northwards over time (Cabrera, Jacobs, and Kadereit 2011). An enhanced diversification took place during the Pliocene, possibly driven by increasing aridity, together with inland migration along palaeodrainage systems especially pronounced in species adapted to coastal conditions (Cabrera, Jacobs, and Kadereit 2011; Kadereit and Freitag 2011).

McDonald (2020) subsequently examined the evolution of chenopods in Australia, proposing several key hypotheses regarding their diversification and migration. He hypothesised that the primary evolutionary spaces for the range expansion and diversification of most chenopod taxa (including Camphorosmeae) were the Yilgarn and Eyre‐Murray centres. These regions, which contain 97% of all Australian chenopod species, are not restricted to the arid zone but represent critical centres for chenopod evolution. He noted that these centres share 43% of all species, indicating widespread initial colonisation from coastlines followed by multiple migrations across these regions. He furthermore identified the inland province of Sandland South (Great Victoria Desert) as an important migration link between the Yilgarn and Eyre‐Murray centres, rather than the coastal Nullarbor Plain, dominated by chenopod vegetation today (albeit with low species diversity). Finally, he also noted that the chenopod abundance in Riverine Deserts and Desert Lakes underscores the influence of niche conservatism, particularly regarding salinity and flooding in the main inland habitat of chenopods.

Despite these valuable insights, both studies suffered from unresolved phylogenies with low levels of support and overall limited taxon sampling. A recent study by Hühn et al. (2024) re‐evaluated the diversification of the Australian Camphorosmeae using an adapted RADseq approach. Sequence data were generated for 104 species from all 12 Australian genera. A modified NGS‐based methodology was used to improve the phylogenetic resolution, identify statistically supported clades and place the phylogeny into a temporal frame. Seventeen statistically well‐supported clades were identified, with their habitat preference demonstrating the influence of landscape change and the emergence of new habitats in arid Australia since the late Miocene, with migration likely following a west‐to‐east pattern of aridification. The Camphorosmeae arrival coincided with significant palaeoclimatic, landscape and biotic changes. Possibly aided by preadaptation of their progenitors to coastal (i.e., saline) environments, Hühn et al. (2024) suggested that Camphorosmeae migrated inland along Riverine Desert landscapes (a habitat type defined by Mabbutt (1988)) formed by changes in palaeodrainage systems in southern and western Australia (Hühn et al. 2024).

Those three primary research hypotheses, proposed by Cabrera, Jacobs, and Kadereit (2011), McDonald (2020) and Hühn et al. (2024), offer significant insights into the evolutionary history of Camphorosmeae in Australia. While sharing common elements, these hypotheses exhibit unique differences that highlight the complex interplay of diverse mechanisms of dispersal, colonisation and adaptation. All of them concur that the ancestors of Australian Camphorosmeae arrived via a long‐distance dispersal event from continental Eurasia during the Middle to Late Miocene. This period marks the beginning of significant climatic shifts, leading to the aridification of Australia. The adaptation of Camphorosmeae to arid and saline environments is a central topic across these hypotheses, suggesting that these plants were pre‐adapted to such conditions before they arrived in Australia. Hühn et al. (2024) and Cabrera, Jacobs, and Kadereit (2011) share similar results, particularly in their biogeographical patterns and diversification trends. Both propose that initial colonisation occurred in the south and west of Australia, followed by expansion across the continent. They also emphasise the Late Miocene and Pliocene as periods of significant diversification, driven by the intensifying aridification. However, they note a slowdown in speciation during the Pleistocene, likely due to habitat contraction and climatic oscillations. Nevertheless, it is important to consider the alternative explanation proposed by Cusimano and Renner (2010), who suggest that such slowdowns in diversification rates may be artefacts of incomplete sampling rather than genuine biological phenomena. This raises the possibility that the observed slowdown in speciation during the Pleistocene in both studies could be influenced by gaps in phylogenetic or fossil data. McDonald's hypothesis introduces the littoral connection hypothesis (Burbidge 1960; Shmida 1985), positing that chenopods initially arrived in the coastal regions, from where they expanded towards inland. This hypothesis underscores niche conservatism, where species retain ancestral ecological characteristics, influencing their migration patterns and habitat preferences. McDonald also explores two scenarios—fragmentation and amalgamation—to explain the development of chenopod diversity in Australia, providing a unique perspective on range expansion and evolutionary centres. The fragmentation scenario involves an initial colonisation and establishment phase with coastal bridgeheads, followed by a landscape spread where chenopods extend their range, particularly towards the west and south coasts, linked through the ancestral Great Victoria Desert. This is followed by two diversification stages, where speciation increases and distinct lineages form in the western Yilgarn and central‐eastern Eyre‐Murray centres of diversity. Conversely, the amalgamation scenario begins similarly with coastal bridgehead formation and expansion but follows with separate provincial expansions and early diversification in the west and east. As aridity intensifies, these centres merge, facilitating species exchange and forming the subcontinental Arid‐Mediterranean group through further range expansion (McDonald 2020).

Each of the three approaches has its methodological considerations and challenges. Cabrera, Jacobs, and Kadereit (2011), for example, highlighted the reliance of molecular data for age estimates in the absence of direct fossil evidence, noting that these estimates are consistent with the fossil record of chenopod‐like pollen in Australia. The oldest chenopod‐like pollen fossils were discovered at the boundary between the Oligocene and Miocene and are the oldest known in Australia (Martin 1981; Christophel 1989; Cabrera, Jacobs, and Kadereit 2011). However, the study suffered from poor resolution of the phylogenetic tree. McDonald (2020), on the other hand, drew most of his conclusions based on the ecology of the species and the landform evolution of individual habitats on palaeoenvironmental and geological evidence but did not fully resolve the phylogenetic history of the taxon in question. This was succeeded only by Hühn et al. (2024), by using a modified RADseq protocol for sequencing. Their study revealed new clades and at least partially overcame the issue of poor phylogenetic resolution, despite the challenges posed by the high proportion of missing data.

Building on these hypotheses, our research aims to integrate their strengths and unique aspects through a phylogenetically based ancestral analysis. Rather than testing each hypothesis individually, we focus on synthesising the insights from these studies to place the evolutionary history of Camphorosmeae into a spatial context and infer its ancestral ecological niche. By using a well‐sampled and resolved phylogeny, we aim to explore the roles of coastal and inland pathways in the dispersal and diversification of Camphorosmeae, emphasising the impact of climatic fluctuations and ecological niche conservatism. To validate these hypotheses regarding the climatic niches, habitat types, evolutionary dynamics and biogeography of Australian Camphorosmeae, mapping habitat type occupations or bioclimatic variables onto the well‐resolved dated phylogenetic tree of Hühn et al. (2024) will reveal clade‐specific shifts, providing insight into how different Camphorosmeae species have adapted to various habitats or responded to climate change. Additionally, biogeographical analyses will help unravel the complex interplay between evolutionary history, geographical distribution and diversification events over time.

Overall, this study aims to deepen the understanding of the evolutionary history of Australian Camphorosmeae by integrating phylogenetic analyses with biogeographical models, climate data and habitat preferences, offering new insights into their distribution and diversification across Australia's diverse landscapes.

## 2 Materials and Methods

### 2.1 Taxon Sampling and Occurrence Data

For this study, the georeferenced distribution points of 159 species, representing all 12 genera of the Camphorosmeae, were extracted from Australia's online species database, the Atlas of Living Australia (ALA), a repository of biodiversity data including records from lodged herbarium specimens (Belbin and Williams 2016; Belbin et al. 2021).

Before analysis, manual cleaning steps were required, which included removing records without coordinates, distribution points outside Australia and around botanical gardens, duplicate coordinates per species and records categorised under taxonomic ranks such as ‘genus’ or ‘family’. To ensure that only naturally occurring distributions were included, we conducted an additional filtering step to exclude any records flagged as ‘cultivated’ in the dataset. Additionally, we cross‐checked the records for incorrect synonymisation relying on plantsoftheworldonline.org (POWO 2024). To relate the present analyses to the most recent dated phylogeny of the Australian Camphorosmeae by Hühn et al. (2024), distribution points of 103 Australian Camphorosmeae species included in the phylogeny, except for *Sclerolaena* sp. *yeltacowie* ineditus due to missing coordinates, were selected (Table S1).

### 2.2 Bioclimatic Variables and Principal Component Analysis

All 19 bioclimatic variables were extracted from WorldClim v.2 (Fick and Hijmans 2017) at a spatial resolution of 2.5 min (~ 4.5 km at the equator), to perform Principal Component Analysis (PCA) aimed at determining the optimal bioclimatic variables for reconstructing their ancestral state based on the dated Australian Camphorosmeae phylogeny. The extent() and crop() methods in R v4.0.2 (R Core Team, 2020), using RStudio v1.2.5042 (RStudio Inc. 2009–2020), were used to obtain bioclimatic data specifically for the Australian habitat. To achieve this, the extent for Australia was defined as, *x*min = 112, *x*max = 154, *y*min = −44 and *y*max = −10. Bioclimatic variable values were extracted for each presence point of the 103 Australian Camphorosmeae. With this dataset, the PCA was carried out to obtain the best bioclimatic variables for further analyses.

To evaluate the proportion of the climatic niche occupied by Camphorosmeae in Australia, we quantified the climatic space of Australia using 406,372 climatic data points. These data points represent the entire climatic space of Australia at a resolution of 2.5 arc minutes. We then performed a PCA to capture the entire range of climatic variation across the continent, allowing us to assess the distribution of Camphorosmeae within this climatic space. After conducting PCA to identify the major axes of variation, we projected the locations of all occurrences from 103 Australian Camphorosmeae species onto the principal component (PC) space. This transformation enabled us to observe and analyse the distribution patterns of Camphorosmeae in Australia within this modified space. In addition, PCA was conducted on the climatic data of the Australian Camphorosmeae. The principal components, capturing the most significant variation, were identified (PC1 and PC2) for the variable correlation plot, with variables exhibiting high loadings used to determine the orientation of the axes. We selected the variables with the highest loading value for PC1 and PC2, respectively, essentially choosing the variables that contribute the most to the variability captured by PC1 and PC2, respectively. Since they were capturing different sources of variability, they were less likely to be highly correlated with each other. Additionally, a pairwise Pearson's correlation coefficient test was done to evaluate collinearity among these variables.

### 2.3 Ancestral State Reconstruction With Key Climatic Variables and Ancestral Habitat Type Reconstruction

To investigate the evolutionary history and ecological adaptations of Australian Camphorosmeae two different reconstructions were made: Ancestral Bioclimatic Reconstruction with the two most relevant environmental bioclimatic variables (Bio05—Maximum Temperature of Warmest Month; and Bio13—Precipitation in the Wettest Month) and Ancestral Habitat Type Reconstruction based on defined habitat types. The dated maximum clade credibility (MCC) tree by Hühn et al. (2024) was used. For the Ancestral Bioclimatic Reconstruction, the mean value of each bioclimatic variable for 103 species was calculated. We treated these characters as continuous and used the ML‐based ‘ace’ function from the ‘ape’ package in R (Joy et al. 2016; Paradis and Schliep 2019) to perform the ancestral state reconstruction. The results were visualised using the ‘contMap’ function from the ‘phytools’ package in R (Revell 2024), which allowed for a more accurate depiction of the evolutionary changes of these continuous traits over time.

Following the publication by Hühn et al. (2024), which inferred the ancestral status of habitat types based on chronological order, this study conducted a formal analysis using ‘Ancestral Habitat Type Reconstruction’ to test their assumptions. To do so, 10 habitat types defined by Mabbutt (1988) and McDonald (2020) were used. The specific habitat types corresponding to the individual species were derived from the work of Hühn et al. (2024). Since habitat types represent discrete categorical variables, the maximum parsimony method was considered appropriate for this ancestral reconstruction. Accordingly, we followed the topology of Hühn et al. (2024) and implemented the reconstruction using Mesquite (v3.81, www.mesquiteproject.org).

### 2.4 Biogeographical Analysis

To investigate the biogeographical history of Australian Camphorosmeae, again, the dated MCC tree by Hühn et al. (2024) was used in the R package ‘BioGeoBEARS’ v1.1.2 (Matzke, 2013) and its dependencies. BioGeoBEARS presents a versatile, likelihood‐based system, designed to characterise the dynamic shifts of branches within phylogenies across discrete biomes over evolutionary time. These biome shifts encompass both anagenetic events, occurring within a single branch, such as dispersal and extinction processes (DEC; Ree and Smith 2008), and cladogenetic events, which transpire at branching points and involve sympatric (BAYAREALIKE; Landis et al. 2013), vicariant (DIVALIKE; Ronquist 1997) and founder‐event speciation processes (DEC + J, BAYAREALIKE+J, DIVALIKE+J; Matzke 2014, Van Dam and Matzke 2016).

A large‐scale analysis based on an almost complete sampling by Hühn et al. (2024) was performed. For the distribution data, 20 Australian phytogeographical subregions were selected following the ranges identified by Ebach et al. (2015). Subregions were chosen for each species only if more than 10% of their occurrence points were present within those areas. Any subregion with ≤ 10% of occurrence points per species was treated as insignificant. This enables the main occurrences of the respective species to be analysed and avoids potential identification biases. The required subregion shapefile from Ebach et al. (2015) was downloaded for this purpose. However, since some species in Camphorosmeae occur very close to the coast, adjustments to the shapefile were necessary. The edges of the shapefile did not always align precisely with the coast, leading to the exclusion of some coastal occurrence points in the initial attempt. Consequently, the shapefile was overlaid on an Australian landscape map via QGIS v3.30 (QGIS, 2024) and the coastal boundaries were extended accordingly. This adaptation allowed the inclusion of many distribution points near the coast in the biogeographical analysis for coastal species such as *Threlkeldia diffusa* R.Br., *Maireana oppositifolia* (F. Muell.) Paul G. Wilson, *M. brevifolia* (R. Br.) Paul G. Wilson, *Sclerolaena uniflora* R. Br. and *Dissocarpus biflorus* (R. Br.) F. Muell.

As a result, there were one to a maximum of four main subregions for most of the species. A table showing this reduction can be found in the supplement (Table S2). Nine subregions in total for 103 species were selected: Central Desert, Central Queensland, Adelaide, Eyre Peninsula, Nullarbor, Southwest Interzone, Southwestern, Eastern Desert and Western Desert. The maximum range size was set to four, as the most widely distributed Australian Camphorosmeae species covered a maximum of four subregions. Six models have been tested (DEC, BAYAREALIKE and DIVALIKE and their +J alternatives). The best‐fit model was chosen by comparing the corrected Akaike information criterion (AICc) and Akaike weight (AICc_wt).

## 3 Results

### 3.1 Data Compilation and Cleaning

A total of 228,875 occurrence points from ALA were compiled (Atlas of Living Australia, 2021; Atlas of Living Australia occurrence download at https://doi.org/10.26197/ala.a4134452‐6171‐4ad7‐8da0‐46682199a2d8. Accessed 30 May 2023). Manual data cleaning reduced the total number of records to 195,758. Distribution points of 103 Australian Camphorosmeae species included in the most recently dated phylogeny by Hühn et al. (2024) were specifically selected for the analyses (Table S1). This results in 178,355 included distribution points.

### 3.2 Bioclimatic Characterisation and Niche Occupancy

**FIGURE 1:**
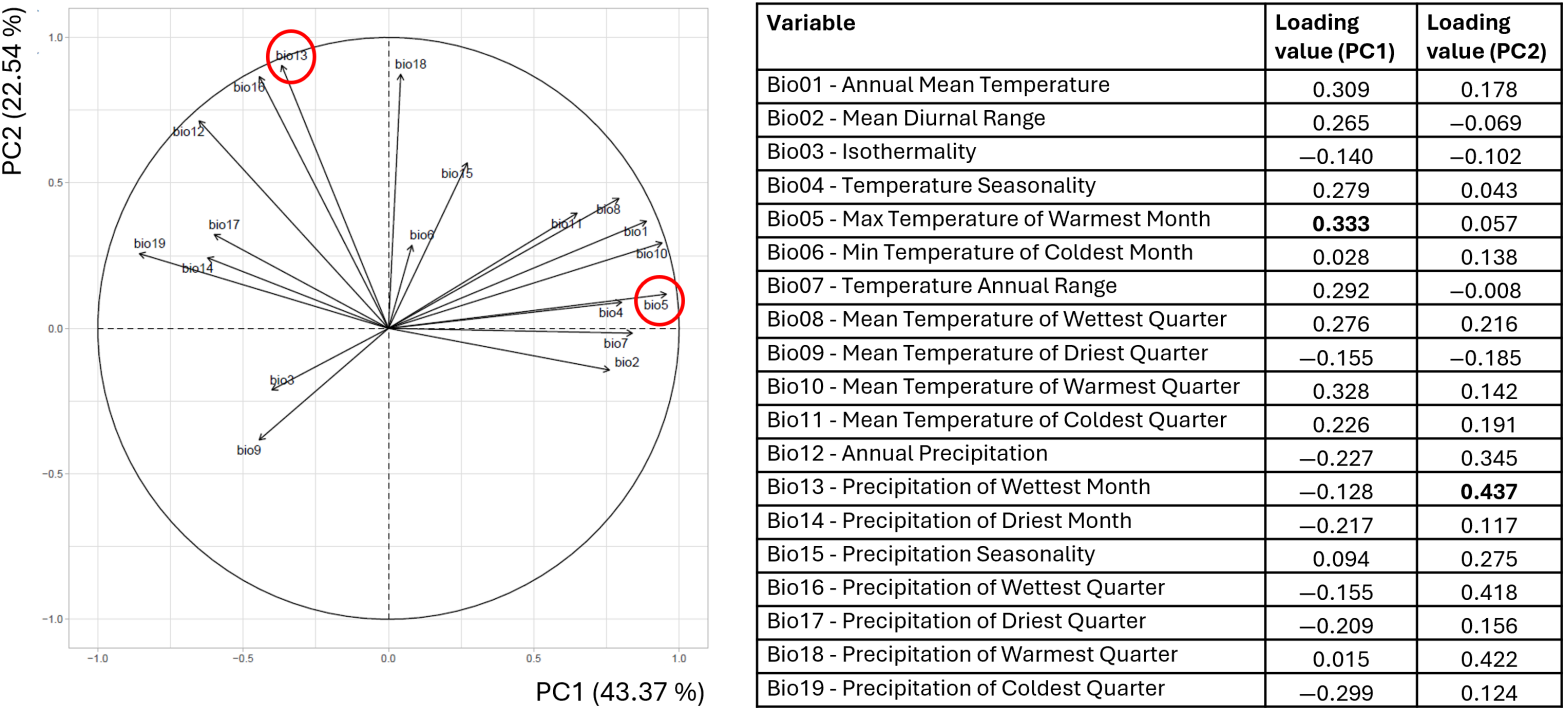
Principal component analysis (PCA) Biplot and Loadings for 19 bioclimatic variables conducted with all distribution points of 103 Australian Camphorosmeae species together. The biplot shows the first principal component (PC1) on the *x*‐axis and the second principal component (PC2) on the *y*‐axis. Each vector represents a bioclimatic variable, with the direction and length indicating its contribution to the principal components. Variables Bio05 and Bio13 are highlighted on the biplot as they have significant contributions to PC1 and PC2, respectively. The table on the right lists the loading values of each variable on PC1 and PC2. The bold values indicate the variables with the highest loadings on each principal component. PCA loadings can be found in Table S3.

This analysis aimed to identify the bioclimatic variables driving the distribution patterns of Australian Camphorosmeae and to assess the proportion of climatic niches occupied by the species within the Australian habitat. Through the Principal Component Analyses (PCA) of 19 bioclimatic variables, significant axes of climatic variation could be identified. The PCA was conducted with all distribution points of 103 Australian Camphorosmeae species together (Figure 1).

The resulting correlation circle, displayed in Figure 1, represented the distribution of 19 environmental variables (Bio01 to Bio19) across the first two principal components, PC1 and PC2, and revealed strong relationships between those variables based on their distance from the centre and orientation relative to each other. Bioclimatic variables located far from the centre and close to each other exhibited strong positive correlations (*r*‐value close to 1), while those far from the centre and orthogonal were not correlated (*r*‐value close to 0). Variables positioned on opposite sides of the centre demonstrated significant negative correlations (*r* close to −1).

**FIGURE 2:**
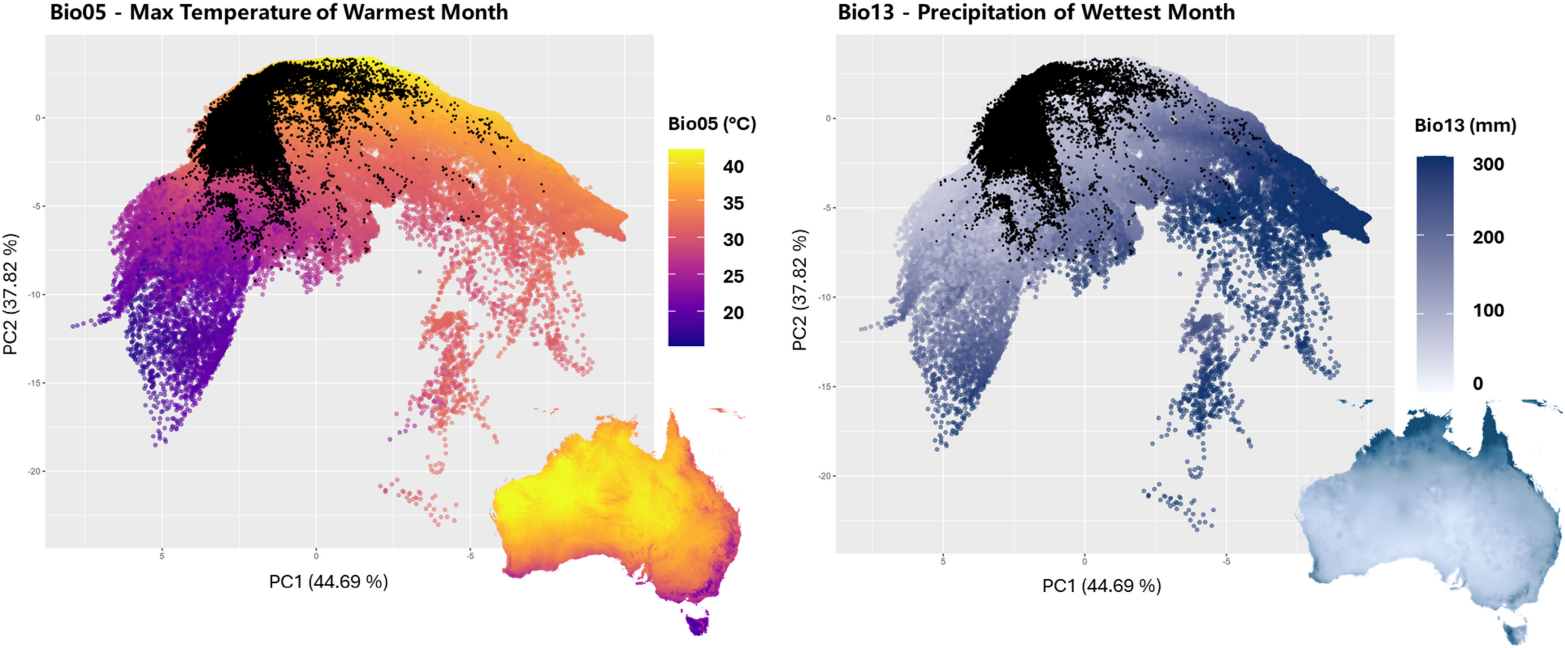
PCA biplots for Bio05 (Maximum Temperature of Warmest Month) and Bio13 (Precipitation of Wettest Month) over Australia's climatic space. Each point represents one of 406,372 locations across Australia, derived from climatic data at a 2.5 arc‐minute resolution. For Bio05 (maximum temperature of the warmest month, °C), points are coloured according to temperature, with a gradient ranging from purple (cooler temperatures) to yellow (warmer temperatures). For Bio13, points are coloured according to the precipitation of the wettest month in millimetres (mm). The colour gradient ranges from light blue (lower precipitation) to dark blue (higher precipitation). Inset maps show the spatial distribution of these bioclimatic variables across Australia. Black dots indicate the distribution points of 103 Australian Camphorosmeae species.

PCA analysis of 103 species together, explained a substantial portion of the variability among all climatic variables, with both PC axes accounting for 65.91% of the total variance (Figure 1). PC1 and PC2 explained 43.37% and 22.54% of the total variance, respectively. Interpretation of the loading values highlighted the dominant climatic axes captured by each principal component. PC1, primarily reflecting the temperature axis, was strongly influenced by variables such as Bio05 (Maximum Temperature of the Warmest Month; loading value of 0.333) and Bio10 (Mean Temperature of the Warmest Quarter; loading value of 0.328). On the other hand, PC2, representing the precipitation axis, was mainly driven by variables including Bio13 (Precipitation in the Wettest Month; loading value of 0.437) and Bio18 (Precipitation in the Warmest Quarter; loading value of 0.422), indicating their substantial contribution to this component. In addition to the correlation circle, the Pearson correlation coefficient test also showed strong correlations between the variables Bio05 and Bio10, and between Bio13 and Bio18. Therefore, only the variable with the highest loading value per PC was selected for further analysis (Bio05 and Bio13). Initially, they were used to project the distributions of Australian Camphorosmeae species onto their PC space (Figure 2).

The climatic niche occupied by the Camphorosmeae species in Australia was evaluated by conducting a PCA on the entire climatic range of Australia. Figure 2 shows the PCA of the climatic range across Australia, based on 406,372 location points for each of the two bioclimatic variables. The PC space, with PC1 and PC2 accounting for 44.69% and 37.82% of the total variance, respectively, highlights the significant contributions of these principal components. These components were used to project the occurrence of 103 Australian Camphorosmeae species onto the PC space. The Camphorosmeae species predominantly occupy areas with high maximum temperatures, as indicated by the clustering of occurrence points in the warmer regions of the climatic space. The species occurrences are concentrated in areas with low to moderate rainfall, with a notable presence in regions experiencing lower precipitation.

**FIGURE 3:**
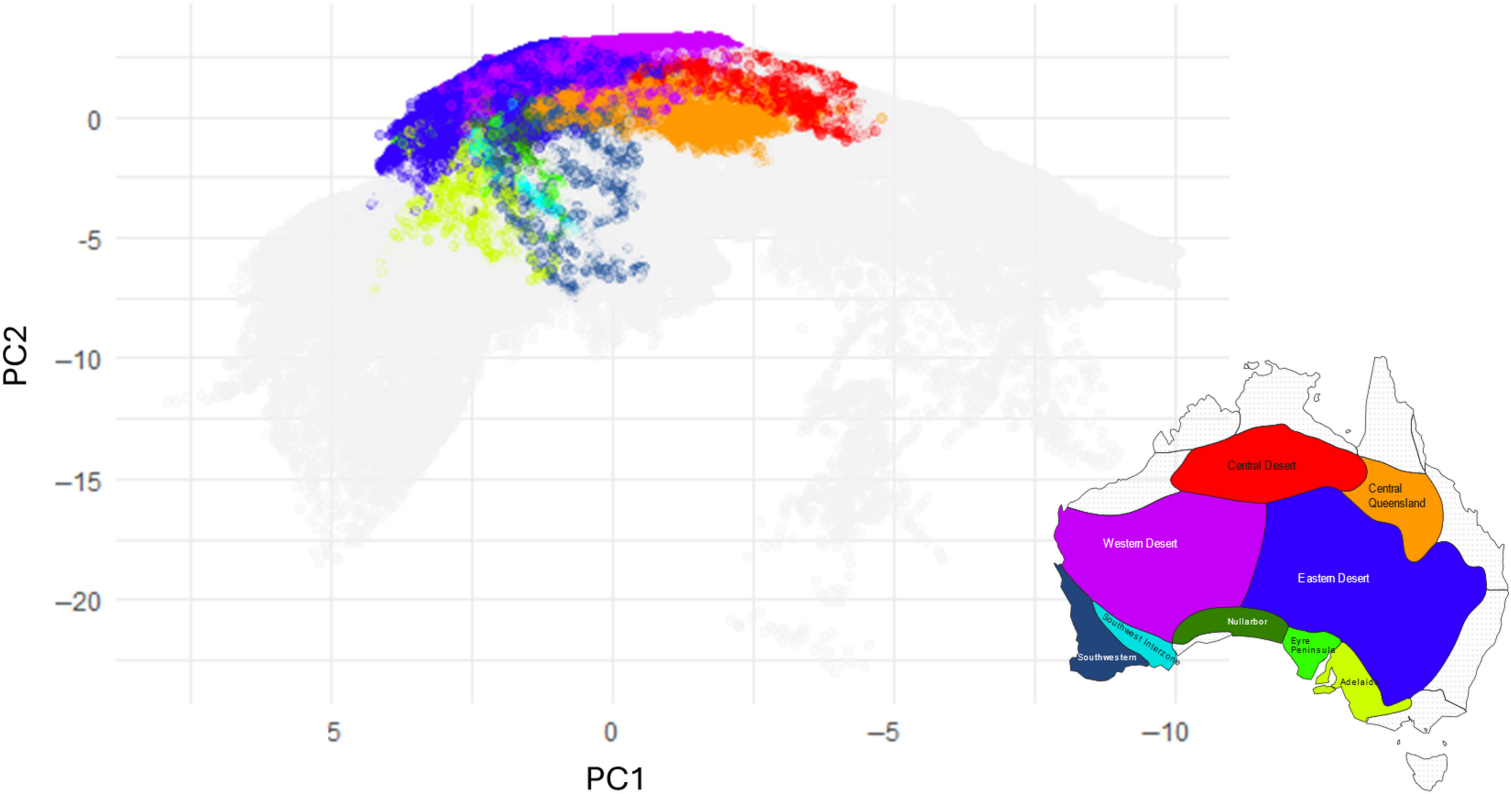
PCA of climate range across Australia. Each point represents one of 406,372 locations across Australia, derived from climatic data at a 2.5 arc‐minute resolution. Coloured dots represent the subregions with more than 10% of the occurrence points per species. Subregions are shown on the map. PCA loadings can be found in Table S3.

Figure 3 represents the principal component (PC) space, with colours representing the subregions of Australia as defined by Ebach et al. (2015). Only subregions with more than 10% of occurrence points per species are displayed. These visualisations provide insights into the main axes of climatic variation and the spatial distribution of climatic conditions across the continent. PC1 explains a significant portion of the variance, capturing a broad climatic gradient across Australia. PC2 represents another important climatic gradient, orthogonal to PC1.

### 3.3 Ancestral Estimation and Transitions

**FIGURE 4:**
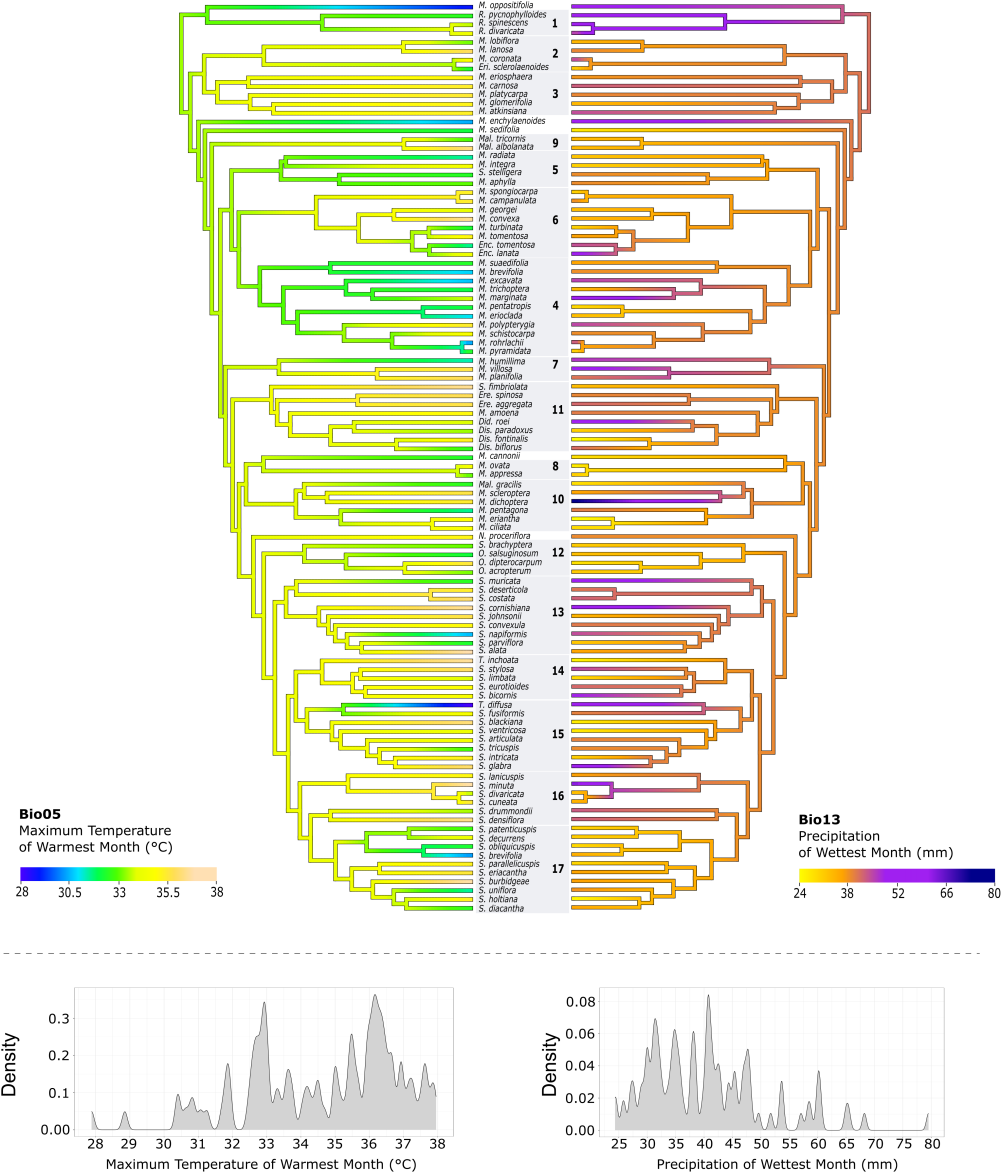
Phylogenetic visualisation of bioclimatic niche reconstruction at ancestral nodes for two environmental variables in the phylogenetic MCC tree of Australian Camphorosmeae. Density plots show the distribution frequency across all clades. Bio05: Branches are coloured by temperature (°C). Bio13: Branches are coloured by precipitation (mm).

**FIGURE 5:**
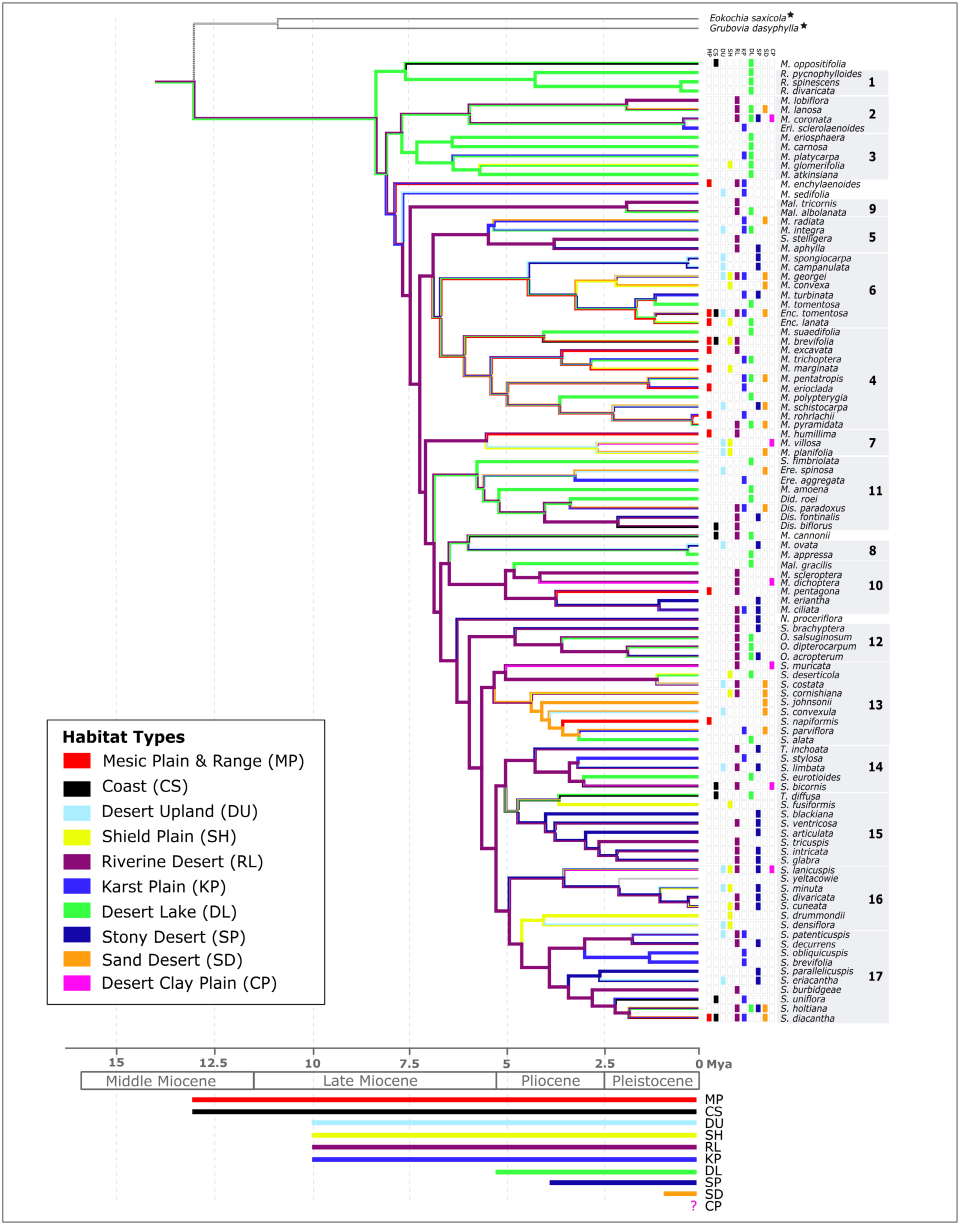
Ancestral Habitat Type Reconstruction for the Australian Camphorosmeae based on the dated MCC tree. It represents the evolutionary relationships among 104 (*Sclerolaena* sp. *yeltacowie* included) Australian Camphorosmeae species with 10 habitat types defined by Mabbutt (1988) and McDonald (2020). Branch colours indicate each node's most likely ancestral habitat type, inferred from habitat reconstruction analyses using Mesquite's maximum parsimony method. The right side of the tree shows the current habitat type distributions for each species based on McDonald (2020). Major clades are labelled from 1 to 17 based on Hühn et al. (2024). The phylogeny is calibrated with that time scale at the bottom, divided into the Middle Miocene, Late Miocene, Pliocene and Pleistocene epochs. The horizontal bars below the time scale show the different habitat types over geological periods based on McDonald (2020). Outgroup species are marked with a star.

The bioclimatic niche evolution of those two selected bioclimatic variables (Bio05 and Bio13) is shown in Figure 4, and the Ancestral Habitat Type Reconstruction in Figure 5. To facilitate the evaluation and discussion of these patterns and results, the same clade numbers as assigned by Hühn et al. (2024) were utilised.

Considering the influence of the two bioclimatic variables, a discernible pattern emerges. Firstly, it is apparent that the ancestor of Australian Camphorosmeae and the ancestors of clades 1–6, 9, as well as *Maireana oppositifolia, M. sedifolia* (F. Muell.) Paul G. Wilson and *M. enchylaenoides* (F. Muell.) Paul G. Wilson preferred lower peak temperatures in the warmest month (Bio05) of under 34°C. Subsequently, a multiple transition in numerous clades towards slightly warmer Maximal temperatures of the warmest month is observed in the descendants, namely Clades 7–8, 10–17, *M. cannonii* (J. M. Black) Paul G. Wilson, and *Neobassia proceriflora* (F. Muell.) A. J. Scott. Interestingly, a subsequent return to lower temperatures occurs in some of these species.

Looking at the precipitation of the wettest month (Bio13), the ancestor of Australian Camphorosmeae likely preferred at least 40 mm of precipitation during the wettest month. Ancestors of clades 1–3 and *M. enchylaenoides* were more likely to be found in slightly wetter areas with precipitation values more than 40 mm during the wettest month, transitioning into marginally drier conditions (≤ 38 mm). Multiple shifts back to areas with wetter conditions can be observed on at least 10 terminal nodes (Figure 4).

The reconstruction of the ancestral habitat types shown in Figure 5 reveals three main ancestral types, with Riverine Desert being the most prominent and spanning the entire ancestral backbone. For clades 1–3, Riverine Desert and Desert Lake can be recognised as ancestral habitats. Riverine Desert and Karst Plain for *M. enchylaenoides* and *M. sedifolia* and Riverine Desert for all other clades (Figure 5).

### 3.4 Biogeographical History

For the BioGeoBEARS analyses using a maximum range of four areas, BAYAREALIKE was the best‐fit model (AICc = 822.7; AICc_wt = 0.68), followed by BAYAREALIKE+J (AICc = 824.5; AICc_wt = 0.28). Both models indicate low rates of dispersal (*d* = 0.013) but notably higher extinction (*e* = 0.13) values.

**FIGURE 6:**
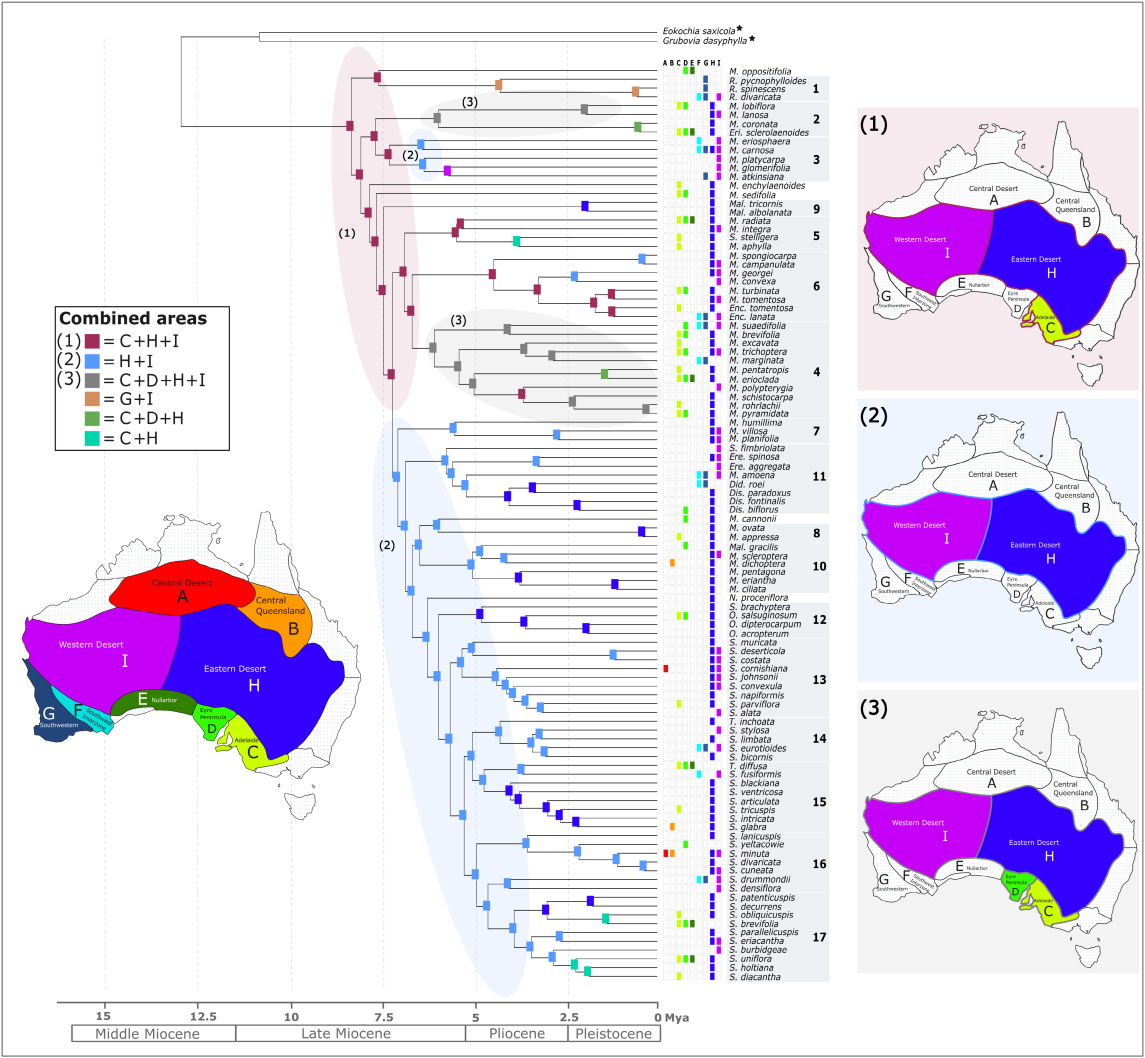
Biogeography analysis on the time‐calibrated phylogenetic MCC tree showing the distribution of Australian Camphorosmeae species across various subregions in Australia over time. Biogeographical analysis was conducted in BioGeoBEARS v1.1.2 with a maximum range size of four. The colour‐coded matrix next to the species names corresponds with the current species distributions. Subregions were chosen only if more than 10% of each species' occurrence points were present within those areas. Squares at nodes represent the ancestral range with the highest probability from the BAYAREALIKE analysis. Colours represent the areas as indicated on the map or combinations of them. The Australian subregion map is based on Ebach et al. 2015. Combined area maps on the right: (1) shows the combination of the subregions C, H and I in dark red, (2) shows the combination of H and I in light blue and (3) shows the combination of C, D, H and I in grey. Outgroup species are marked with a star.

The biogeography analysis highlights two main combinations of subregions within the phylogenetic backbone as potential ancestral areas (Combination 1 and 2). There is a 47.75% probability that the ancestor of clades 1–6 and 9, along with species *Maireana oppositifolia, M. enchylaenoides* and *M. sedifolia* , inhabited Adelaide, Eastern Desert and Western Desert (C + H + I, Figure 6). During the end of the Late Miocene, the ancestors of Clade 3 as well as of Clades 7, 8 and 10–17, along with species *M. cannonii* and *Neobassia proceriflora*, show a significant presence in the Eastern and Western Desert (H + I, Figure 6) regions, with a probability range of 19%–23% for Clade 3 and 60.32% for the rest. Dispersal events to the Eyre Peninsula (D) occurred for clades 2 and 4. These events are represented by the C + D + H + I combination (3) with probabilities of 34.05% and 55.28% at the crown, respectively, contemporaneously during the end of the Late Miocene.

Only during the Pliocene and Pleistocene areas such as the Southwestern (G) in combination with the Western Desert (I) were inhabited. This is evident for Clade 1 at the beginning of the Pliocene. In addition, the Southwestern Interzone (F) was occupied more recently by some species like *Roycea divaricata* Paul G. Wilson, *M. eriosphaera* Paul G. Wilson, *M. carnosa* (Moq.) Paul G. Wilson, *Enchylaena lanata* Paul G. Wilson, *M. suaedifolia* (Paul G. Wilson) Paul G. Wilson, *M. marginata (*Benth.) Paul G. Wilson, *M. amoena* (Diels) Paul G. Wilson, *Didymanthus roei* Endl., *Sclerolaena eurotioides* (F. Muell.) A.J. Scott, *S. fusiformis* Paul. G. Wilson and *S. drummondii* (Benth.) Domin. Additionally, several young species (*Sclerolaena obliquicuspis* (R. H. Anderson) Ulbr, *S* *. brevifolia* (Ising) A. J. Scott, *S* *. uniflora* , *S. holtiana* (Ising) A. J. Scott and *S. diacantha* (Nees) Benth.) from clade 17 migrated into the Adelaide region (C + H, Figure 6) during the early Pleistocene.

## 4 Discussion

Australia's complex palaeoclimatic history has profoundly influenced the evolution and distribution of its flora, including the Camphorosmeae. The evolutionary history of Camphorosmeae in Australia has been the subject of extensive research over the past few decades, with particular emphasis on the integration of morphological, molecular and biogeographical data. The main hypotheses put forward by Cabrera, Jacobs, and Kadereit (2011), McDonald (2020) and Hühn et al. (2024) offer complementary yet distinct perspectives on the biogeographical patterns and processes driving the diversification and distribution of this group. Building on this previous work and integrating biogeographical analyses, climatic niche evolution and habitat type shifts, we reached a more elaborate understanding of the evolution of Camphorosmeae in Australia.

### 4.1 Climatic Influence on Evolution and Distribution of Australian Camphorosmeae

Our study shows significant environmental gradients influencing the occurrence of Camphorosmeae species (Figure 1). The first principal component, driven by Maximum Temperature of Warmest Month (Bio05) and Mean Temperature of the Warmest Quarter (Bio10), explains 43.37% of the variance. This suggests that temperature extremes are crucial in determining and limiting the distribution of these species. The second principal component, linked to Precipitation in the Wettest Month (Bio13) and Precipitation in the Warmest Quarter (Bio18), accounts for 22.54% of the variance, indicating the importance of precipitation minimum and seasonality.

The Camphorosmeae species occupy predominantly areas with high maximum temperatures during the warmest month, as indicated by the clustering of occurrence points in the warmer regions of the climatic space. The maximum temperature in the warmest month for the Australian Camphorosmeae ranges from 27.9°C to 38.0°C, emphasising the adaptation to high temperatures. These temperatures fall within the range observed in the semi‐arid and desert biomes of Australia, where temperatures frequently exceed 35°C during summer, though the upper range of 40°C or more is more characteristic of the central desert regions (Geoscience Australia 1994). Additionally, their distribution in areas of low to moderate precipitation during the wettest month (24.3–79.5 mm) highlights their adaptability to different levels of aridity, with a notable presence in regions experiencing moderate rainfall (37–52 mm). It is important to note that these precipitation levels refer specifically to the wettest month within the distribution range of Camphorosmeae, not to the wettest month in Australia overall. This precipitation range is consistent with the definition of the semi‐arid biome in Australia, where annual rainfall is less than 250–500 mm, and monthly wet season precipitation typically varies within this range (Geoscience Australia 1994; Huang et al. 2016). The exclusively annual genus *Grubovia*, exhibits distinct climatic preferences that closely match those of its Australian relatives. Our analysis of the distribution of *Grubovia* species reveals a preferred maximum temperature in the warmest month (Bio05) ranging from 23.2°C to 27.9°C. This range is lower than the range of the Australian Camphorosmeae, but still overlaps, indicating an adaptation to relatively high temperatures. Regarding precipitation during the wettest month (Bio13), *Grubovia* species thrive in regions with 42.0 mm to 53.3 mm of precipitation. This range is within the wider range of 24.3 mm to 79.5 mm observed for Australian Camphorosmeae. It corresponds to the moderate precipitation conditions of 30–50 mm where the Australian species are most abundant. Thus, while *Grubovia* occupies a slightly cooler and more consistently moderate rainfall niche compared to Australian Camphorosmeae, both clades show strong adaptability to environments characterised by high temperatures and variable rainfall, particularly moderate conditions.

Looking at the ancestral climatic preferences of the Australian Camphorosmeae, we see that they favoured warmest month temperatures up to 34°C and areas with precipitation during the wettest month of at least 52 mm. This suggests that the ancestors of these two sister lineages were already adapted to this type of precipitation regime. The milder and lower the temperatures, similar to those preferred by *Grubovia*, furthermore suggest that the ancestors initially thrived in cooler climates. However, it is important to acknowledge that reconstructing ancestral climatic preferences based on extant taxa is controversial in the literature (Cunningham, Omland, and Oakley 1998; Cunningham 1999; Vanderpoorten and Goffinet 2006), especially given the significant climatic changes since the Pliocene, including cycles of re‐wetting and dry/wet, hot/cold periods during the Pleistocene. Despite this uncertainty, it appears that over time, the lineage that became the Australian Camphorosmeae adapted to the changing climate within Australia since the end of the Late Miocene.

### 4.2 Habitat Evolution

Reconstruction of ancestral habitat types may reveal critical ecological transitions that facilitated the adaptation and diversification of Camphorosmeae species. Our analysis shows distinct clusters of points in principal component (PC) space, indicating unique climatic conditions in different subregions (Figure 3). This reflects the ecological versatility and/or specialisation of these species, suggesting that they have evolved to occupy different climatic niches.

**FIGURE 7:**
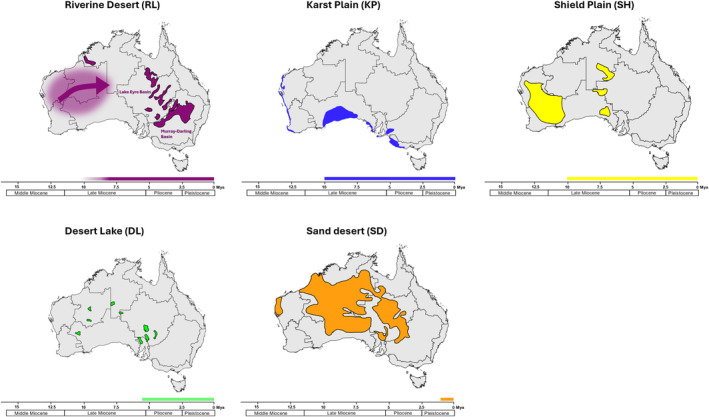
The five important habitat types of the Australian Camphorosmeae. Riverine Desert (RL), Karst Plain (KP), Shield Plain (SH), Desert Lake (DL) and Sand Desert (SD). Each map shows the geographical extent of a particular habitat type and its evolution over geological periods from the Middle Miocene to the Pleistocene based on Mabbutt (1988) and McDonald (2020). The arrow in the Riverine Desert map indicates the distribution through time. Coloured habitat types are based on Mabbutt (1988) and Webb, Grimes, and Osborne (2003).

Our results indicate that the diverse range of habitats occupied by Australian Camphorosmeae highlights significant ecological diversity within the group. The habitat type ‘Riverine Desert’ spans the entire backbone of the phylogeny, strongly suggesting it to be the ancestral habitat type of the Australian Camphorosmeae. This habitat type, characterised by open vegetation and arboreal growth along floodplains and river channels with mild to moderate salinity and variable soil types, likely emerged in the Middle to Late Miocene when palaeovalleys ceased to exist (Figure 7; Van de Graaff et al. 1977; McDonald 2020). Riverine Lake habitats have been present in Western Australia since the Miocene gradually shifted with the progressive cessation of the palaeodrainage systems (Kershaw, Martin, and Mason 1994; Hühn et al. 2024). As these systems slowly disappeared, the river systems transformed into desert river channels, and the floodplains began to dry out, creating the ‘Riverine Desert’ habitat. This habitat type, which is well suited to species adapted to high temperatures and arid to semi‐arid regions, expanded eastwards due to the west‐to‐east gradient of declining precipitation and increasing aridity that occurred during the Late Miocene and Pliocene (Martin 2006). It is consistent with the adaptations observed in many Australian Camphorosmeae species. This demonstrates that ‘Riverine Desert’ landscapes played a significant role as a migration corridor, further facilitating the spread and diversification of these species, as suggested by McDonald (2020) and Hühn et al. (2024) and is supported by our analysis. Certain clades strongly associate with other specific habitats, such as the ‘Shield Plain’ for clades 6 and 16, ‘Desert Lake’ for clades 1–3, 8 and 11 and ‘Sand Desert’ habitats for clade 13. This suggests that habitat specialisation has played a crucial role in the evolutionary divergence of the lineage. The adaptability of Camphorosmeae to semi‐arid and arid environments prevalent in Australia is reflected by the strong association between specific clades and particular habitats, indicating that habitat shift might have been one key driver of diversification within this group (Figure 5). The ‘Shield Plain’ habitats are non‐saline and contain a variety of xerophytic plant species (McDonald 2020). *Sclerolaena drummondii* and *S. densiflora* (W. Fitzg.) A. J. Scott are two examples of a habitat shift from the regularly inundated ‘Riverine Desert’ to the highly exposed rocky ‘Shield Plain’ habitat. The ‘Desert Lake’ habitat is characterised by a strong salinity gradient, ranging from the bare salt crust to the slightly salty edges with halophytic vegetation (Mabbutt 1988). The species present there (e.g., most *Maireana* species), have a high‐temperature tolerance, allowing them to thrive in this environment, while those adapted to moderate amounts of precipitation are likely to be found on the less saline edges where conditions are less extreme. The very young ‘Sand Desert’ habitat, which formed less than a million years ago during the Late Pleistocene (Fujioka and Chappell 2010), is characterised by hummock grasslands, tall shrublands or open woodlands, and generally lacks salinity. This is the case except in areas where thin sand sheets cover older landscapes inhabited by glycophytes that are more competitive in the absence of salinity (McDonald 2020; Flowers, Galal, and Bromham 2010; Waisel 1972). It is important to mention that these habitat types are flexible in time and location and are very similar in their overall climatic conditions. They have changed continuously with changes in geography and climate, and the boundaries of the arid and semi‐arid areas have changed since the end of the Miocene in response to fluctuations in temperatures (Smith 2013).

### 4.3 Biogeography and Historical Processes

The conducted large‐scale biogeographical analysis identifies key dispersal and vicariance events that have shaped the current distribution of Camphorosmeae species. The overlap and separation of habitat types and occupied subregions highlight the diversity of climatic niches occupied by these species, reflecting historical biogeographical processes (Figures 3, 5, 6 and 7). These findings provide insights into the evolutionary pressures and ecological dynamics that have influenced the distribution patterns of the Australian Camphorosmeae.

The combination of the Western Desert, Eastern Desert and Adelaide geographical subregions (sensu Ebach et al. 2015) is identified as the ancestral range of Australian Camphorosmeae. Throughout the evolutionary timeline, particularly from the Late Miocene to the Pliocene, this region served as the core habitat for many Australian Camphorosmeae. The combination of Western and Eastern Desert, the so‐called outback, occurs at several points in the phylogenetic tree. In particular, it is retrieved as the ancestral region for clades 3, 7–8 and 10–17. The persistence of this combination may highlight the climatic stability and continuity of these deserts throughout the Late Neogene. Alternatively, the role of the age and gradual expansion of the arid/semi‐arid biome could be considered. The arid climate initially developed in northern and central Australia and progressively expanded southwards as the continent drifted northwards. This implies that the Western and Eastern Desert regions might have been among the first arid habitats to form, serving as key refuge areas for Camphorosmeae. The later expansion into the Eyre Peninsula, observed in clades 2 and 4, could reflect this gradual southwards spread of arid conditions. The Eyre Peninsula's open vegetation during the Early to Middle Pliocene, as reported by Kershaw, Martin, and Mason (1994), supports this scenario of biome expansion rather than just climatic stability.

Southwestern Australia has been identified as a significant source of lineages that dispersed into arid regions, particularly the Eremaean zone. This region has acted as a centre of diversification for several other plant groups. The asymmetric dispersal of *Calytrix* from southwestern Australia to the Eremaean region highlights the role of southwestern Australia as a key source area for arid‐adapted flora (Nge et al. 2022). Similarly, Goodeniaceae species repeatedly dispersed from southwestern Australia into the arid central and southeastern regions of Australia, especially after the Miocene aridification (Jabaily et al. 2014). In previous studies, it was assumed that the southwest part of Australia could be the area of origin of the Camphorosmeae (Cabrera, Jacobs, and Kadereit 2011). However, more recent analyses, including those by Hühn et al. (2024), challenged this view. Lamont and He (2017) proposed that the Mediterranean‐type climate (MTC), which currently exists in southwestern Australia, likely originated in northwestern Australia around 30 Ma. As the Australian continent drifted northwards, the MTC shifted southwards towards its current location. This climatic shift suggests that the ancestors of Australian Camphorosmeae may not have initially dispersed directly to the southwest but rather into northwestern Australia, taking advantage of more uniform precipitation patterns at the time. As the continent aridified and the MTC established further south, the lineage likely followed this climatic gradient, dispersing progressively towards the southwest. This challenges previous assumptions about the migration of Camphorosmeae, suggesting a more complex dispersal history driven by large‐scale climate shifts. Similar counterarguments to the southwest origin hypothesis have been presented in studies on other Australian plant groups. For example, McLay, Bayly, and Ladiges (2016) argues that for taxa such as *Hakea*, the southwestern region may not represent a true centre of origin but could instead reflect an artefact due to a methodological bias arising from phylogenetic analyses. Their work questions whether divergence patterns attributed to southwestern Australia could be more accurately explained by broader continental processes rather than regional isolation. Nge et al. (2024) further demonstrated that in groups like *Cryptandra* (Rhamnaceae), spine evolution and the diversification of these taxa occurred in response to environmental changes across multiple regions, not just in the southwest. In their earlier work, Nge et al. (2022) also found that *Calytrix* species showed biogeographical disjunctions between eastern and southwestern Australia, indicating that these plants underwent complex evolutionary processes far beyond the southwestern region alone. Thus, while the littoral connection hypothesis still provides a plausible explanation for the observed distribution of chenopods (McDonald 2020), suggesting that they maintained their ecological preferences over time and directed their spread from coastal regions into the interior deserts, it is likely that this process involved multiple dispersal phases. Beginning in the north and moving southwards in conjunction with the shifting climatic zones, this process seems to align more closely with continental‐scale dispersal mechanisms observed in other Australian plant taxa. Ultimately, the idea of southwestern Australia as a singular centre of origin must be reconsidered in light of these broader biogeographical studies that illustrate more complex evolutionary and dispersal histories across the Australian continent.

The recovery of the ‘Riverine Desert’ as an ancestral habitat type adds another layer of complexity. While this habitat type supports the idea of a Western origin due to the presence of certain taxa, it appears to contradict the broader biogeographical patterns observed (Figure 7). The data suggests that while there may be taxa in the western part of the Western Desert, the main ancestral range remains more centrally located between the Western and Eastern Deserts and Adelaide, due to the presence of many Late Miocene/Early Pliocene lineages and the greatest diversity of taxa (Figures 5, 6, 7). The dynamic environmental conditions described by Lamont and He (2017), particularly the southward migration of the MTC, may have played a crucial role in shaping these distribution patterns, influencing both the dispersal and adaptation of Camphorosmeae species across different arid and semi‐arid areas of Australia.

### 4.4 Migration and Local Adaptation

This complex dispersal history of the Camphorosmeae mirrors broader patterns of plant migration and adaptation observed across Australia's arid zones, where changing climates have shaped biodiversity in significant ways (Crisp and Cook 2013; Weston, Jordan, and Keith 2017). Plants in arid regions of Australia have developed a variety of complex evolutionary strategies to survive, avoid and persist in hot and water‐limited environments (Norton, Malinowski, and Volaire 2016). These strategies include processes of adaptation, in which plants change their structures or functions to better suit their environment (Dörken, Ladd, and Parsons 2020), and migration, in which they disperse seeds to new areas that offer suitable conditions. The evolution of plant groups in Australia's Eremaean Zone involves vicariance, pre‐adapted immigration and in situ adaptation (Cauz‐Santos et al. 2024). For example, *Eucalyptus* and *Calytrix*, both from the Myrtaceae family, are examples of vicariance (Cauz‐Santos et al. 2024; Nge et al. 2022; Martin 2006). An example of pre‐adapted immigration, however, is the genus *Ptilotus*, which was already adapted to thrive in arid environments (Hammer et al. 2021). In situ adaptation illustrates the evolution of traits specialised for arid conditions within the Eremaean zone. An example of in situ adaptation in Australia is the persistence of *Banksia* (Proteaceae) species in response to changing climatic conditions, facilitated by adaptive genetic variation within populations (He et al. 2016). The Australasian grass flora reflects similar processes. The migration and adaptation of grasses is influenced by factors such as climate stability and the suitability of new environments. The spread of grasses to Australia was facilitated by their pre‐adaptation to dry and high light that is, UV conditions, allowing them to outcompete native species (Bryceson et al. 2023). This migration was enhanced by broad land bridges that existed during the Last Glacial Maxima due to lower sea levels (Bryceson and Morgan 2022). Dispersal and vicariance have also driven diversification in other plant lineages across Australia's arid zones. *Acacia* (Fabaceae), for instance, demonstrates a wide range of habitat adaptations within the arid zone. Research by Ladiges, Ariati, and Murphy (2006) and Renner et al. (2021) reveals that *Acacia* species exhibit diverse adaptations to varying climatic conditions. Analysis by Ladiges, Ariati, and Murphy (2006) indicates that the differentiation of *Acacia* species among regions, such as Arnhem and the northwest semi‐arid regions, suggests ancient biogeographical divisions that potentially date back to the Cenozoic era. The study highlights how *Acacia* species have radiated into different habitats within the arid zone, reflecting a complex interplay between migration and local adaptation. It demonstrates the range of habitats that *Acacia* has colonised and adapted to, contrasting with the more specialised habitat preferences observed in Camphorosmeae. *Cryptandra* (Rhamnaceae) diversified through several vicariant events, followed by in situ diversification with little exchange between regions, since the diversification of this genus was negatively affected by the spread of aridity (Nge et al. 2024). This contrasts with *Hakea* (Proteaceae), which underwent frequent biome shifts, showing a similar dispersal pattern to Camphorosmeae, by diversifying in southern Australia and dispersing into other biomes across the Australian continent, including arid regions (Cardillo et al. 2017). Similarly, *Callitris* (Cupressaceae) diversified in response to increasing aridity from the Oligocene onwards, exhibiting adaptations to extreme aridity, such as high embolism resistance (Larter et al. 2017). These patterns suggest that ecological pressures, such as those faced by Camphorosmeae in saline and xeric environments, play a key role in shaping the flora of Australia's arid zone.

In our study, we observe several transitions from arid ecologies to areas with higher precipitation during the wettest month. Consequently, new traits are required for species to successfully invade these habitats. According to the dated phylogeny, these in situ transitions are recent and involve comparatively young species and clades. This pattern of habitat transition is not unique to our study; for example, research by Hammer et al. (2021) on *Ptilotus* also documents a similar shift from arid to wetter environments in some clades, highlighting a broader trend of adaptive trait evolution associated with changing precipitation patterns. Additionally, it is important to note that our study shows also examples of recent dispersal events into arid zones, which should be considered in the context of these findings.

The two species‐rich genera of the Australian Camphorosmeae, *Maireana* and *Sclerolaena* are known to prefer environments such as lake systems and saline areas. These species are likely to be influenced not only by edaphic factors such as soil type but also by the hydrology of the area, which is highly variable. There have been significant changes during the Pliocene, particularly around the Lake Eyre system, affecting the extent of lakes and saline environments (Habeck‐Fardy and Nanson 2014).

Our formal analyses clarify and detail patterns previously hypothesised, revealing both migration and local adaptation in different species. For example, the *Roycea* group in clade 1, which is one of the early divergent lineages, is nowadays widespread in western Australia, mostly in the subregions Southwestern (*R. pycnophylloides*, *R. spinescens* and *R. divaricata* ), Western Desert and Southwest Interzone ( *R. divaricata* ), with Western Desert (I), the Eastern Desert (H) and Adelaide (C) as the ancestral areas (Figure 6). The ancestors of these three species are likely to have originated in the Riverine Desert habitat and remained in the area while the habitat shifted eastwards and other habitats arose or changed (Figure 7). The Desert Lake habitat, for example, began to form in the Pliocene, of western Australia. The age of the current habitats in which *Roycea* occurs do not match the age of the lineage. This suggests that *Roycea* did not migrate with the Riverine Desert habitat, which originated in the west of Australia (Cabrera 2007) and shifted eastwards but instead adapted to the Desert Lake habitat in west Australia (Figures 5 and 7). Another example is the two species *Sclerolaena drummondii* and *S. densiflora* that occur in the Shield Plain habitat. That habitat originated in the Yilgran Block in Western Australia and central Australia in the Arunta Block (Mabbutt 1988) during the Late Miocene. Both species adapted to the Shield Plain habitat in place, while the Riverine Desert habitat shifted eastwards.

Previous hypotheses suggested that the Southwestern Interzone (F) and the Southwestern (G) habitat types might be ancestral, but this analysis indicates otherwise. Focusing on the Riverine Desert, discussing its age, distribution and role as a connecting habitat is crucial. These rivers around the Lake Eyre and Murray‐Darling Basin, which are ephemeral and flow for only 3–4 months following seasonal rainfall, follow biogeographical patterns. This demonstrates a balance between niche conservatism and ecological flexibility. Species that retained the Riverine Desert niche migrated east with the habitat, while others that either were ecologically broader and not dependent on the Riverine Deserts, or managed to adapt to the new habitats could remain in the west.

Travelling further east from the arid centre, seasonality becomes more pronounced, and precipitation decreases. In the far southeast, the Great Dividing Range, including the Australian Alps, creates a significant rain shadow, reducing rainfall further inland. This geographical barrier has shaped the climate across the region, limiting moisture in areas to the west of the range. Ancestral lineages that migrated southeast, such as the clades 9 and 12, as indicated by branches showing precipitation values under and equal to 38 mm during the wettest month (Figure 4), adapted to these increasingly arid conditions. The Riverine Desert has long been considered a connecting habitat, and our findings confirm this notion. Hühn et al. (2024) initially hypothesised this and our formal analysis, using ancestral character state reconstruction, supports and extends the conclusions by Hühn et al. (2024). Some species that migrated south or towards the coast of Australia like *Maireana enchylaenoides*, *M. marginata* , *Enchylaena lanata*, *Didymanthus roei* or *Threlkeldia diffusa* have branches showing precipitation equal to or greater than 52 mm during the wettest month (Figure 4). Although the ancestors were adapted to survive in areas with precipitation equal to or greater than 52 mm, the descendant species occurred in areas with less than 52 mm precipitation during the wettest month. These mentioned species are presumably more competitive in regions with a precipitation of 52 mm or more in the wettest month.

In examining the interplay between migration and adaptation in Australian Camphorosmeae in Australia's dynamic habitats, it becomes clear that distinguishing between the two processes is complex by nature. Habitats and distribution areas of species are both changing constantly, making it difficult to clearly separate migration from local adaptation. In most cases, a mixture of both processes is at work, with one occasionally dominating the other. This observation aligns with the evolutionary histories of many other Australian plant lineages, where aridification, biome shifts and convergent evolution have shaped the flora of Australia's arid zones. Consequently, it is reasonable to question the need for a clear distinction between migration and adaptation especially in areas that are connected.

### 4.5 Challenges and Further Work

Studying the general biology of the Australian Camphorosmeae is challenging due to several factors, including Australia's wide and often remote landscapes, which can hinder fieldwork and sample collection, resulting in sampling gaps. Additionally, the complex geological history of the Australian continent, characterised by tectonic activity, climatic fluctuations and sea level changes, has influenced the distribution and diversification of plant species over millions of years (Dettmann 1994). Furthermore, the Camphorosmeae tribe comprises species with different ecological requirements and dispersal abilities (Cabrera 2007), further complicating biogeographical analyses.

The phylogenetic analysis also presents challenges with temporal constraints, especially concerning the Sand Desert habitat. The discrepancy between the estimated age of the sand desert and the migration patterns of Camphorosmeae species, particularly in clade 13, which inhabits the Sand Desert, is shown in Figure 5. This suggests either an error in estimating the age of the habitat, or multiple migrations into the Sand Desert over time. These taxa might have had different ecologies and only recently migrate into the Sand Desert. Evidence, particularly from groups such as *Triodia* spp., suggests that the Sand Desert habitats are relatively recent (Mabbutt 1988; Grigg 2009). The branch length extending back 5 to 4 million years complicates the interpretation, as introducing temporal constraints into the biogeographical model may not significantly affect the state shift estimates. The habitat types, such as Karst Plain, Shield Plain or Desert Lake span multiple geographical regions, making it difficult to perform precise biogeographical reconstructions, a problem exacerbated by the constant shifting of those regions. Similarly, clades 1–3 and the species *M. oppositifolia* show Desert Lake as an ancestral habitat dated to the Late Miocene, whereas the estimated age of these habitats based on palaeogeographical evidence appears to be the Early Pliocene in Western Australia and Late Pliocene in Central Australia (McDonald 2020). If we accept these estimated habitat ages, only the Riverine Desert could be the ancestral habitat due to its age. However, this raises questions about the true ancestral status given the unresolved backbone of the phylogeny. In addition to these biogeographical uncertainties, it is worth noting that inferring ancestral climatic preferences from extant taxa presents additional complexities (Cunningham, Omland, and Oakley 1998; Cunningham 1999; Vanderpoorten and Goffinet 2006). The evolutionary trajectory of these lineages has been shaped undoubtedly by significant climatic changes since the Pliocene, including periods of warming, cooling and fluctuating precipitation patterns during the Pleistocene. These environmental changes would have affected the ecological niches of ancestral taxa, suggesting that caution is required when interpreting their past climatic adaptations. Thus, while the preferences of current species can provide clues, they may not fully represent the conditions experienced by their ancestors. The diverse range of habitats occupied by Camphorosmeae species, particularly their association with saline environments and fluctuating hydrological conditions (Cabrera, Jacobs, and Kadereit 2011; Hühn et al. 2024), suggests that edaphic factors and hydrology may influence their distribution greatly. This variability, particularly around systems such as Lake Eyre, highlights the need for finer scale analyses and potentially more nuanced biogeographical coding to capture the dynamic environmental changes over the past 8 million years.

Data limitations are another challenge. The partly unresolved backbone and the absence of 56 species (36 *Sclerolaena*, 15 *Maireana*, one *Dissocarpus*, one *Malacocera*, one *Neobassia* and two *Osteocarpum* species) from the phylogeny limit the scope of the analysis. Although the current phylogeny represents an improvement over previous datasets and does not exhibit significant sampling bias, including these species in future analyses could refine our understanding of their evolutionary and biogeographical patterns. After conducting an additional analysis to account for the missing 56 species by filtering the most important subregions (i.e., areas with more than 10% of occurrences per species), three further subregions (Eastern Queensland, Great Sandy Desert Interzone and Southeastern) were identified to be important (Table S2, Figure S1). It may be that the inclusion of ecologically highly specialised species such as *Sclerolaena hostilis* (Diels) Domin would be a valuable contribution to a more comprehensive phylogenetic framework, as this species only occurs in the Great Sandy Desert Interzone with a 100% occurrence rate. In future work, we additionally aim to further explore the role of soil variation in shaping diversification rates within Camphorosmeae, particularly by incorporating more precise clade‐specific sampling probabilities, additional soil quality parameters and methods such as target enrichment for improved genetic sampling. While preliminary analyses have not yielded conclusive results, more refined data and approaches may help to clarify the influence of soil characteristics on diversification patterns.

All these challenges highlight the complexity of studying the evolutionary and biogeographical history of Camphorosmeae species in Australia. Future research should address these limitations by improving phylogenetic resolution through methods such as target enrichment or genome skimming, conducting finer scale habitat and biogeographical analyses and including currently missing species to provide a complete picture of their evolutionary dynamics. The distinction between migration and adaptation can be effectively studied through the combined use of population genetics and ecological niche modelling. These methods provide complementary insights into the processes that drive species distribution and evolution.

## 5 Conclusion

The study of Camphorosmeae in Australia reveals a vibrant evolutionary and biogeographical history shaped by climate change and habitat diversification. The combination of morphological, molecular and biogeographical data has provided insights into the evolutionary dynamics of this lineage, highlighting the influence of temperature and precipitation on species distributions. The ancestral adaptation to warm and moderately humid climates allowed the migration and local adaptation of Camphorosmeae to different habitats, in particular, the Riverine Desert, which played a crucial role in their diversification. Despite advances in understanding, challenges such as incomplete sampling and complex geological histories remain, requiring further research to refine phylogenetic frameworks and biogeographical models. Overall, this research highlights the interplay between ecological flexibility and niche conservatism in shaping the biodiversity of Australian Camphorosmeae.

## Author Contributions

**Jessica A. Berasategui:** conceptualization (lead), data curation (lead), formal analysis (lead), visualization (lead), writing – original draft (lead), writing – review and editing (lead). **Anže Žerdoner Čalasan:** conceptualization (supporting), writing – original draft (supporting), writing – review and editing (supporting). **Gudrun Kadereit:** conceptualization (supporting), formal analysis (supporting), funding acquisition (lead), project administration (lead), writing – original draft (supporting), writing – review and editing (supporting).

## Conflicts of Interest

The authors declare no conflicts of interest.

## Supporting information


**Figure S1.** Occurrence of excluded species.

**Table S1.** Coordinates of all species downloaded from ALA.

**Table S2.** Percentage of occurrence points per species across subregions.

**Table S3.** PCA loadings.

## Data Availability

The data that support the findings of this study are uploaded as Supporting Information.
