# Supplementary figures and images for: Biogeography of Australian Camphorosmeae and Diversification in Climatic Space and Across Arid Habitat Types
Source: Ecol Evol. 2024 Nov 14;14(11):e70558. doi: 10.1002/ece3.70558 (PMC11563692; doi:10.1002/ece3.70558)

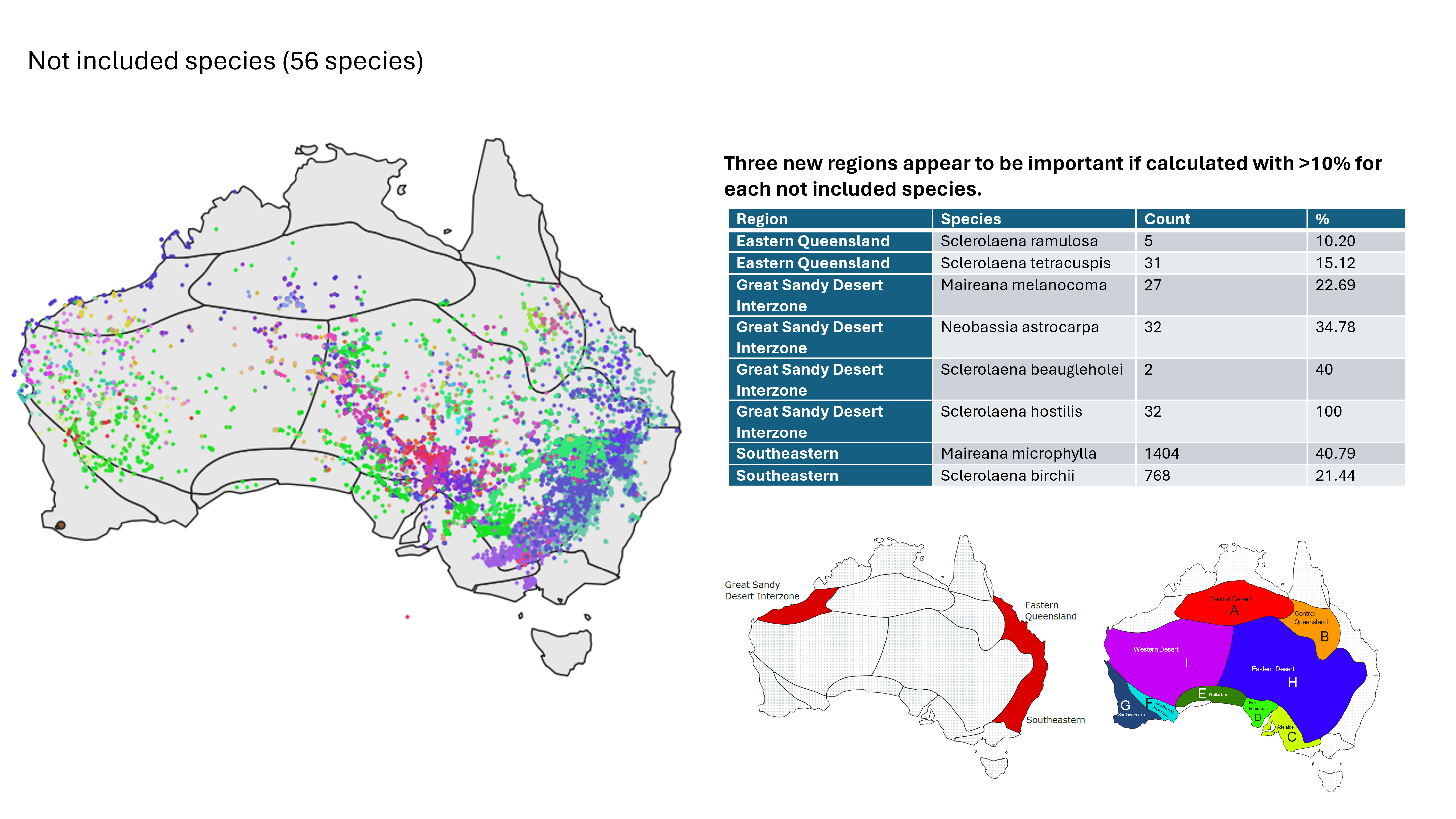

Supplement: Supplementary file 1 — Figure S1. Occurrence of excluded species. [file ECE3-14-e70558-s002.png]
